# Hyperglycemia enhances group B *Streptococcus* pathogenicity by impairing TLR2 expression and chemotactic response in the human placenta

**DOI:** 10.3389/fimmu.2025.1610381

**Published:** 2025-07-17

**Authors:** Rodrigo Jiménez-Escutia, Arumi Villafuerte-Pérez, Donovan Vargas-Alcantar, Karina Martínez-Garfias, Samara Rodríguez-Flores, Pilar Velázquez-Sánchez, Amaury Fortanel-Fonseca, Rodrigo Zamora-Escudero, Marcela Islas-López, Ismael Mancilla-Herrera, Lorenza Díaz, Verónica Zaga-Clavellina, Andrea Olmos-Ortiz

**Affiliations:** ^1^ Departamento de Inmunobioquímica, Instituto Nacional de Perinatología Isidro Espinosa de los Reyes, Mexico City, Mexico; ^2^ Posgrado en Ciencias Biológicas, Universidad Nacional Autónoma de México, Mexico City, Mexico; ^3^ Posgrado en Ciencias de la Salud, Escuela Superior de Medicina, Instituto Politécnico Nacional, Mexico City, Mexico; ^4^ Departamento de Ginecología y Obstetricia, Hospital Ángeles México, Mexico City, Mexico; ^5^ Ginecología y Obstetricia, Hospital Ángeles Lomas – Universidad Nacional Autónoma de México (UNAM), Naucalpan, Mexico; ^6^ Subdirección de Investigación Biomédica, Instituto Nacional de Perinatología Isidro Espinosa de los Reyes, Mexico City, Mexico; ^7^ Departamento de Biología de la Reproducción, Instituto Nacional de Ciencias Médicas y Nutrición Salvador Zubirán, Mexico City, Mexico; ^8^ Dirección de Investigación, Instituto Nacional de Perinatología Isidro Espinosa de los Reyes, Mexico City, Mexico

**Keywords:** gestational diabetes mellitus, glucose, monocytes, NK cells, MCP-1, TLR-2, *Streptococcus agalactiae*, MIP-1 beta

## Abstract

**Introduction:**

Elevated glucose levels during pregnancy disrupt placental structure, signaling, and cellular interactions, impairing its immune response. In mothers with gestational diabetes mellitus (GDM), *Streptococcus agalactiae* (Group B *Streptococcus*, GBS) is the second leading cause of bacterial infections. GDM is also linked to altered chemokine profiles in maternal serum and placenta tissue. However, the impact of hyperglycemia on placental immune responses to bacterial infections remains poorly understood. This work aimed to evaluate cytokine and chemokine production, as well as chemotactic responses, in the placenta following GBS infection under hyperglycemic conditions.

**Methods:**

Human villous explants from term, normoevolutive pregnancies were cultured with 5, 10 or 50 mM glucose, and subsequently infected or not with GBS. Bacterial growth and adherence to villous tissue, syncytial disruption, cytokine and chemokine mRNA expression and secretion, leukocyte chemotaxis using intervillous blood mononuclear cells (IVMC), and TLR-2 expression at both mRNA and protein levels, were evaluated.

**Results:**

Under high glucose conditions, GBS showed increased proliferation and invasiveness, while villous explants presented evidence of syncytial barrier degradation. Also, placental TNF-α, MCP-1, and MIP-1β were induced by GBS infection. However, the dual challenge of high glucose and infection reduced the above inflammatory markers’ gene and protein synthesis. GBS infection enhanced IVMC migration compared to uninfected groups, but the combination of GBS and hyperglycemia led to a reduced migration of IVMC, particularly monocytes and NK cells. TLR-2 placental expression was also downregulated by this dual challenge.

**Conclusion:**

At the placental level, hyperglycemia attenuates the immune response against GBS infection, promoting syncytial disruption, bacterial growth, and tissue colonization. The combined stimulus of hyperglycemia and GBS resulted in reduced placental expression of TLR-2, TNF-α, MCP-1, and MIP-1β, thereby impairing the chemotaxis of IVMC, monocytes, and NK cells. This dysregulated immune response may compromise bacterial clearance and placental integrity, favoring pathogen persistence. Our findings suggest a potential mechanism by which hyperglycemia increases susceptibility to GBS-associated complications, offering novel insight into the interplay between metabolic and infectious stressors at the maternal-fetal interface.

## 1 Introduction

Gestational diabetes mellitus (GDM) is defined as chronic hyperglycemia during pregnancy in the absence of previously diagnosed diabetes. This metabolic disorder affects around 15% of pregnant women worldwide (1), and increases the risk of multiple maternal and fetal complications, both in the short and long term (2–4). The persistent elevation of blood sugar levels during this critical period disrupts numerous immune responses in the placenta, leading to increased production of cytokines and chemokines, enhanced bacterial proliferation, and a heightened susceptibility to infectious diseases (5–7). In this sense, women with GDM face a 30 to 60% higher risk of developing persistent and recurrent infections, including urinary tract infections (UTI) and cervicovaginal infections compared to healthy patients (8–11). In mothers with GDM, *Streptococcus agalactiae* (Group B *Streptococcus*, GBS) is the second leading cause of bacterial infections (9) and one of the major contributors to severe neonatal infections, such as sepsis and meningitis (12, 13).

During pregnancy, the placenta is critically affected by elevated glucose levels, which alter its morphology, signaling, and cellular interactions, ultimately compromising its response to infection (14, 15). A crucial initial event in the response to infection is the recognition of pathogens by various pattern recognition receptors, such as the Toll-like receptors (TLRs). The placenta expresses all TLRs throughout gestation (16), including TLR-2 and TLR-6, which heterodimerize to detect GBS bacteria. Both *in vitro* and *in vivo* studies have demonstrated that exposure to high glucose concentrations upregulates the activity and synthesis of TLR-2 (17–19). In addition, we previously reported that high glucose levels promote GBS proliferation and attachment to villi syncytia (5). We also observed that GBS infection increased the placental secretion of TNF-α and IL-6, as expected; however, the combined challenge of GBS and high glucose resulted in a diminished production of these cytokines. We hypothesize that the attenuated inflammatory response by hyperglycemic environment prevent excessive inflammation, but it simultaneously limits an important innate defense mechanism against infection, increasing the host’s vulnerability to pathogen virulence.

In addition to cytokines, chemokines are also important glucose-dependent targets at the feto-maternal interface. Chemokines are key mediators of the immune response, playing a critical role in attracting both innate and adaptive immune cells, especially in response to infection. GDM has been associated with an altered chemokine profile, characterized by elevated serum levels and increased placental synthesis of CCL-2 (MCP-1), CXCL-1 (GROa), CXCL-8 (IL-8), CXCL-9 (MIG), CXCL-10 (IP-10), CXCL-12 (SDF-1), and CXCL-16 (SRPSOX) (20–24). However, there is still limited information regarding how hyperglycemia influences the placental secretion of chemokines in response to an infectious challenge, such as GBS. Therefore, this study aims to investigate the impact of hyperglycemia and GBS infection on the chemotaxis of immune cells to the placental villi and the production of villous chemokines. Additionally, we investigate the underlying mechanisms responsible for the altered chemotactic response in the context of hyperglycemia and infection. Specifically, we assessed TLR-2 expression as a potential modulator of this response. This data may help to elucidate why patients with GDM are more susceptible to developing GBS infection during pregnancy.

## 2 Materials and methods

### 2.1 Ethics statement

This protocol received approval from the Biosafety, Ethical, and Research Committee of the Instituto Nacional de Perinatología (INPer, code numbers 2023-1–6 and 2018-1-152), as well as from the Hospital Ángeles México (HAMx-MPVS-2023-1), Hospital Ángeles Lomas (HAL 366/2020), all located in Mexico City. Before the cesarean section, written informed consent, as outlined in the Declaration of Helsinki, was obtained voluntarily from each mother.

### 2.2 Reagents

DMEM low glucose culture media (#BIO-L0066-500) and newborn calf serum (#S0750-500) were from Biowest (Riverside, MO, USA). Monohydrated dextrose (#JT1910-1) and D-mannitol (#2554-01) were acquired from JT Baker- Fisher Scientific (Mexico City, Mexico) and were diluted in deionized water to 1M solution, filtered, and preserved at 4 °C. Dextrose (D-glucose) was supplemented to DMEM media to obtain a glucose curve of 5 mM (control glucose), 10 mM and 50 mM (high glucose concentrations). Mannitol was also supplemented in culture media at 45 mM (osmotic control). Lipoteichoic acid (LTA, #L215) from *Staphylococcus aureus* was from Sigma-Aldrich (St. Louis, MO, USA). *Streptococcus agalactiae* Lehmann and Neumann was obtained from the American Type Culture Collection (ATCC) #27956. (Rockville, MD, USA). Blood agar (#7242) was obtained from MCD Lab (Mexico). Sterile defibrinated ram blood (#1600 FC) was from Dibico (Mexico).

### 2.3 Inclusion and exclusion criteria

We collected complete placenta specimens from healthy term (37–41 weeks) pregnant women carrying a single fetus, scheduled for cesarean section at the mentioned hospitals in section 2.1.

We excluded from the study patients who had experienced urinary or cervicovaginal infections during the last trimester of pregnancy, those with allergies to penicillin or streptomycin, who had undergone vaginal delivery, with multiple pregnancies, or those with any documented metabolic or endocrine diseases. Additionally, patients with hyperglycemia during surgery (fasting blood glucose > 95 mg/dL) were excluded. Samples indicating recent infection (positive IgM) after rapid SARS-CoV2 antibody testing (placental blood drop) were also eliminated. Cesarean sections were clinically indicated based on medical records for reasons such as breech presentation, cephalopelvic disproportion, previous uterine surgery, history of myomectomy, or the personal choice of the mother.

### 2.4 Microbiological analysis of samples

To ensure tissue sterility, cotyledon explants were processed by standard microbiological testing methods to detect aerobic and facultative anaerobic microorganisms. Only the data from samples with known sterility were analyzed.

### 2.5 Culture of villous explants

The procedure for isolation and culture of villous explants has been described elsewhere (25). Briefly, clean cotyledons were cut into small explants of 3 to 5 mm^3^, and kept in a Petri dish with saline and medium. For the experiments, explants were incubated in 24-well plates with low glucose DMEM supplemented with 10% newborn calf serum, 1% sodium pyruvate, 100 U/mL penicillin, and 100 µg/mL streptomycin, under standard conditions (37 °C, 95% relative humidity and 5% CO_2_).

Villous explants were incubated for 48 hours in supplemented media adjusted to glucose 5, 10, or 50 mM based on previous experiments developed in trophoblasts and placental tissue to mimic normal, and hyperglycemic environments associated with GDM or diabetes (5, 7, 26, 27). Additionally, some explants were cultured with 5 mM glucose and 45 mM mannitol as an osmotic control, given that mannitol is a non-metabolized polyol.

At the end of the experiments, culture media were recovered and kept at -40°C for subsequent use in migration assays or analysis by the multi-analyte flux technique or ELISA. Villous explants were stored in formalin for Brown-Hopps staining, in TRIzol for RNA isolation, or RIPA buffer supplemented with protease inhibitors for the quantification of TLR-2 protein.

### 2.6 GBS infection of villous explants

After 48 hours of incubation with the treatments described in section 2.5, the explants were infected with GBS (1x10^5^ CFU/mL) for 8 hours. Infection was performed in normoglycemic media (i.e. 5 mM) without antibiotics. Therefore, the bacteria were not exposed to high glucose.

At the end of the infection period, bacterial growth was quantified using the plate dilution technique, and the culture media were collected for cytokine and chemokine quantification using the multi-analyte flux technique. For the plate count, 100 μL of the supernatants were collected, and serial dilutions were performed in saline solution + 0.05% Tween 80. Each dilution was plated in triplicate (10 μL) on blood agar plates, which were incubated for 24 hours at 37°C. After incubation, β-hemolytic activity was corroborated, and the number of colonies grown in each dilution was counted. The experiment was discarded if any abnormal colony morphology was observed. The final count was expressed as CFU/mL/gram of tissue.

### 2.7 Brown-Hopps stain for GBS-infected villous explants

The tissue was embedded in paraffin blocks, and sections were cut using a microtome. The sections were then placed on slides and dewaxed. The slides were immersed in a 1% crystal violet solution for two minutes. Gram’s iodine (the mordant) was added for 5 minutes, followed by rinsing with distilled water. The slides were destained with an acetone-alcohol solution for 10 seconds. Next, 0.5% basic fuchsin was applied for five minutes, and the slides were rinsed again. A 0.1% picric acid solution was added until a slight yellow color appeared, and the slides were quickly rinsed with xylene. Finally, the stained slides were coverslipped.

Images were obtained using a Carl Zeiss Lab A.1 microscope equipped with an AxioCam Erc5s camera (Carl Zeiss, Inc., Thornwood, NY, USA) at 10X magnification. Digital processing of the images was performed with Zen 2.3 (blue edition) software (Jena, Germany). Syncytial disruption was quantified in three slides per treatment across three independent GBS-infected experiments. The physical separation of syncytia from the villous tree was recorded by three independent observers using the ImageJ 1.54g program.

### 2.8 Secretion of cytokines and chemokines by multi-analyte flux assay

After thawing the media, the secretion of cytokines (IL-1β, INF-α2, INF-γ, TNF-α, IL-6, IL-10, IL-12p70, IL-17a, IL-18, IL-23 and IL-33) and chemokines (MCP-1, RANTES, IP-10, ENA-78, Eotaxin, GRO-α, IL-8, I-TAC, MIG, MIP-1α, MIP-1β and MIP-3α) into the culture medium was quantified using commercial LegendPlex™ kits from BioLegend (#740809 and #740985, respectively. San Diego, CA, USA). The assays were performed according to the manufacturer’s instructions. The concentration of each analyte was normalized per gram of wet tissue.

### 2.9 Gene expression of IL-1β, IL-6, IL-8, MCP-1, TNF-α and TLR-2

After infection, the tissue was placed in TRIzol and homogenized using a polytron. It was then centrifuged at 28,000 g for 30 seconds, and the supernatant was collected. The RNA was extracted using the commercial Direct-zol RNA mini prep kit from Zymo Research (#R2050). In all cases, the amount and quality of RNA were estimated spectrophotometrically at 260/280 nm (Nanodrop. Thermo), and a constant amount (500 ng) of RNA was reverse transcribed using the commercial Maximum First Strand cDNA Synthesis Kit from Thermo-Scientific (#K1642). The qPCR amplifications were performed in identical conditions for all genes, using the commercial kit LigthCycler^®^ TaqMan^®^ Master Mix from Roche (#04535286001), loading 1 μL of cDNA. GAPDH was used as house-keeping gene to normalize gene expression. The cDNA was then submitted to standard qPCR conditions using the LightCycler 480 Instrument (Roche Diagnostics, Mannheim, Germany). The primers were synthesized by Integrated DNA Technologies. The sequence of primers and the hydrolysis probes are shown in **Supplementary Table 1**.

### 2.10 Isolation of intervillous blood mononuclear cells

With the assistance of surgical tools, the chorioamniotic membranes and umbilical cord were removed. Cuts were made on the maternal side of the placenta using a scalpel blade, and the placenta was transferred to a sterile bag with the maternal side facing downward. The placenta was kept in the bag for 10 minutes to allow intervillous blood to efflux by gravity. Blood was collected with a sterile transfer pipette and placed into heparin tubes. The heparinized blood was transferred to 50 mL conical tubes and diluted 1:3 with 1X Hank’s balanced solution, supplemented with 10 mM HEPES and 34.2 mM NaHCO_3_.

Next, a 15 mL bed of Lymphoprep™ (STEMCELL Technologies #07851) was placed in another 50 mL conical tube. Then, 30 mL of diluted blood was gently added to the Lymphoprep bed. This tube was centrifuged for 40 minutes at 400 g at room temperature. The ring containing IVMC was then recovered. The cells were washed with 1X Hank’s solution and centrifuged at 550 g for 10 minutes. To eliminate erythrocyte contamination, the cells were incubated for 5 minutes in erythrocyte lysis buffer (NH_4_Cl [155 mM], KHCO_3_[10 mM], and EDTA [0.1mM]). After 5 minutes, the reaction was stopped by adding 1X Hank’s solution, and the cells were centrifuged at 550 g for 10 minutes. The resulting pellet was washed two more times with 1X Hank’s solution. The IVMC were then resuspended into RPMI-1640 medium and maintained at 37°C with 5% CO_2_ until used in the functional migration assay.

### 2.11 Migration assay

After isolation of IVMC, cells were labeled with the fluorochrome CellTracker™ Green CMFDA at a 1:1000 dilution (Invitrogen #C2995). The cells were incubated in RPMI medium for 45 minutes at 37°C with 5% CO_2_ in the dark. After incubation, the cells were centrifuged for 5 minutes at 550 g, and three wash cycles with Hank’s solution were performed. The cells were then resuspended in RPMI-1640 medium.

The Boyden chamber model was used with a 5 µm porous membrane. 20% newborn calf serum served as a positive chemoattraction control. 600 μL of villous-conditioned media was placed in the lower chamber as the chemotactic agent, and 500,000 IVMC in 300 μL of RPMI medium were placed in the upper chamber. The migration assay was performed for two hours at 37°C with 5% CO_2_. Afterward, 150 μL from the lower chamber were collected in duplicate and placed in a 96-well plate for reading on a fluorescence spectrophotometer (Synergy 2 microplate reader, Biotek) at 492/517 nm. A calibration curve was constructed with a 7-point fold serial dilution, ranging from 125,000 to 1,953 cells. A control well of spontaneous migration, with fresh DMEM media, was also included to account for gravity-driven movement. The calculation of migrating cells was determined by subtracting the number of spontaneous migration cells from the total cells in the lower chamber.

### 2.12 Phenotyping of migrant cells

In parallel to the previous section, after 2 hours of the IVMC migration assay, the supernatant from the lower chamber was collected and centrifuged at 400 g for 5 minutes. The cells were then labeled with titled volumes of fluorochrome-conjugated antibodies: anti-CD3-FITC, anti-CD16-PE, anti-CD56-PE, anti-CD19-APC (BD Biosciences #340500), and anti-CD14-PE-Cy7 (BD Biosciences # 663195). The labeled IVMC were incubated at room temperature for 15 minutes, protected from light.

Following incubation, the cells were fixed with 250 μL of 1X FLS solution (BD FACS™ Lysing Solution 10X concentrate. BD Biosciences #349202) and incubated for an additional 10 minutes, also protected from light. Afterward, the cells were washed with phosphate-buffered saline (PBS) and centrifuged at 400 g for 5 minutes. The supernatant was discarded, the cells were resuspended in 100 μL of PBS, and the samples were analyzed using a flow cytometer or stored at 4°C, protected from light, for up to 3 days. The FACSAria™III cytometer (BD Biosciences) was used, and 25,000 events were analyzed per sample. Unique events in the mononuclear cell region were selected and filtered based on CD14+, CD3+, CD19+, CD16+CD56+ or CD3+CD16+CD56+ expression for the detection of monocytes, T, B, NK, and NKT cells, respectively. **Supplementary Figure 1** shows the analysis for the batch with selection strategy, parameters, and representative plots.

### 2.13 TLR-2 ELISA

After 8 hours of infection with GBS, the villous explants were collected and placed in RIPA lysis buffer with protease inhibitors (Roche #11836170001). Samples were then stored at -40°C until mechanical lysis. The samples were lysed using a polytron (OMNI International, Tissue Master 125), and centrifuged at 2850 g for 10 minutes at 4°C. The supernatant was collected, and the protein concentration was quantified by the Bradford method. TLR-2 levels were quantified using an ELISA kit from Invitrogen (#EH459RB) according to the manufacturer’s instructions. Each sample was adjusted to 25 μg of protein in 100 μL per well. Detection range was from 0.328–80 ng/mL. For detection, 100 μL TMB (3,3′,5,5′-Tetramethylbenzidine) was used as the substrate, and 50 μL of 0.2N H_2_SO_4_ was added as the stop solution. Samples were read at 450 nm without correction.

### 2.14 Statistical analysis

All statistical analyzes were performed with GraphPadPrism 10.1.1 software (GraphPad Software Inc). A p-value of less than 0.05 (p < 0.05) was considered a statistically significant difference. Using the Shapiro-Wilk test, the distribution of the data was determined as normal or non-normal, and consequently the parametric or non-2parametric statistical test was chosen for comparison between groups. The statistical test and the *post-hoc* used are indicated in the figure captions.

## 3 Results

Thirty-six placentas were processed and analyzed for the complete set of experiments herein. Clinical data of mothers and newborns are presented in **Table 1**. All patients were normotensive, with a pre-gestational BMI < 30 kg/m^2^, and the newborns had normal weight, length, and head circumference adjusted to sex and gestational age.

**Table 1:** Clinical data of mothers and newborns.

Clinical Parameter	Mean ± SD n= 36	Range min - max
Maternal age (years)	33 ± 3	24 – 38
Pre-gestational BMI (kg/m^2^)	23.6 ± 3.1	19.6. – 29.2
Number of pregnancies	2 ± 1	1 - 5
Gestational age (weeks)	38.1 ± 0.6	37.0 – 39.2
SBP (mm Hg)	113 ± 7	96 - 128
DBP (mm Hg)	72 ± 7	60 - 90
Maternal weight gain (kg)	9.7 ± 3.1	4.0 – 20.0
NB weight (kg)	3.00 ± 0.39	2.4 – 3.91
NB weight centiles	49 ± 28	10 - 97
NB length (cm)	49.0 ± 1.5	46.0 – 52.0
NB length centiles	64 ± 24	18 – 98
NB head circumference (cm)	34.5 ± 1.2	32 – 37.5
NB head circumference centiles	74 ± 24	18 – 98
APGAR 1 min	8.5 ± 0.5	7 - 9
APGAR 5 min	9.0 ± 0.2	8 - 10
Placenta weight (g)	498.9 ± 95.9	300 – 685.9
NB sex (male/female) (%/%)	18/18 (50%/50%)	–

APGAR, acronym for the score system of the physical signs of the newborn (Activity, Pulse, Grimace, Appearance, and Respiration). BMI, body mass index. DBP, diastolic blood pressure. SBP, systolic blood pressure. NB, newborn. Placenta weight was registered without fetal membranes and the umbilical cord. Sex- and gestational age- specific centiles for weight, length and head circumference were calculated with the newborn size calculator of the INTER GROWTH-21st Project, available online (43, 44).

Villous explants were cultured under high-glucose conditions, and, as expected, the pro-inflammatory response was confirmed by an increased secretion of TNF-α, IL-1β, and IL-6 (**Supplementary Figures 2A–C**). Additionally, the basal cytokine secretion in response to mannitol suggests that the inflammatory response was specifically attributed to glucose metabolism rather than high osmolar pressure.

### 3.1 Hyperglycemia increased GBS counts and disrupted the syncytial barrier

**Figure 1:**
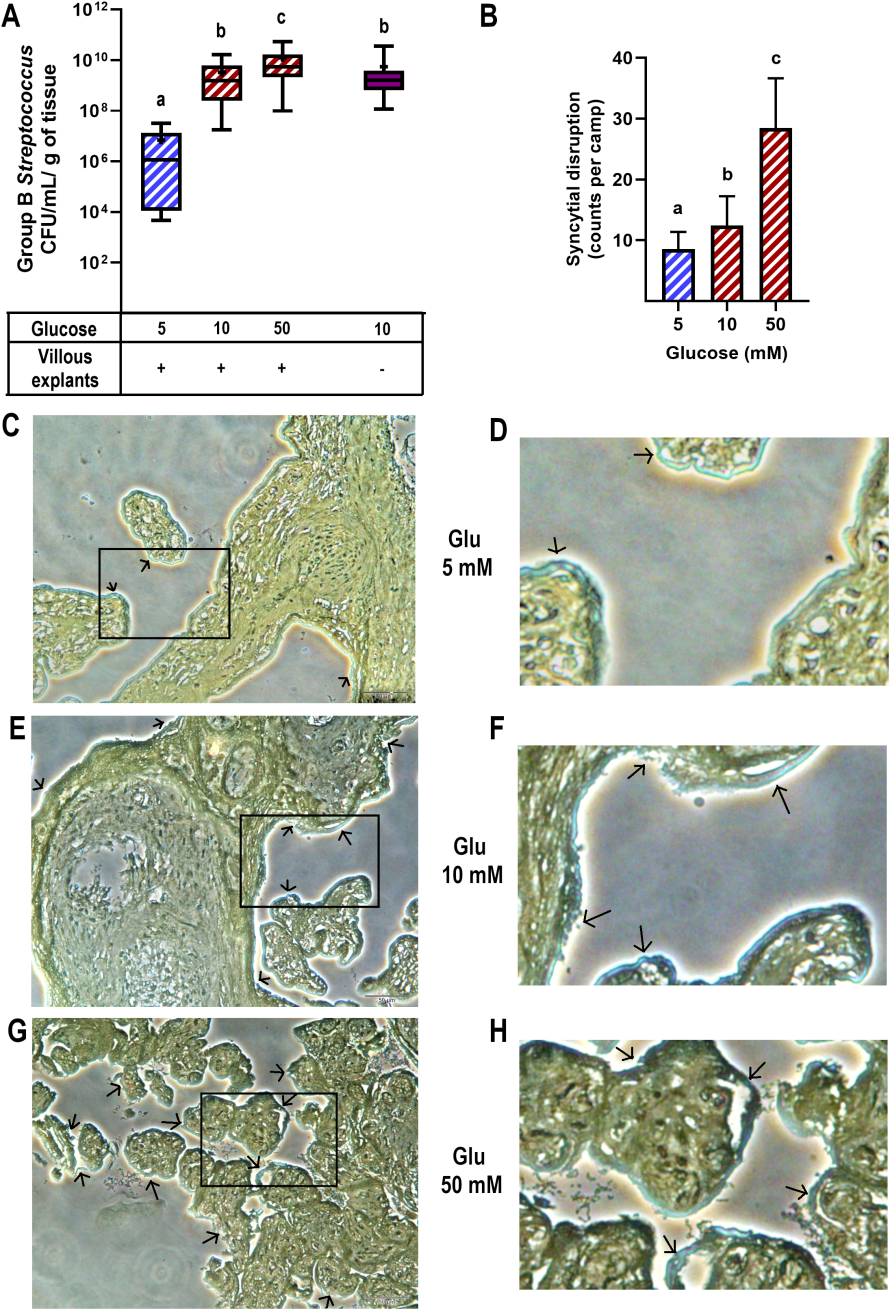
High glucose levels promote GBS proliferation and syncytial disruption in cultured villous explants. Villous explants were exposed to media containing glucose (5, 10 or 50 mM) for 48 h, followed by infection with GBS (1x10^5^ CFU/mL) for 8 h An aliquot of the media was used to assess bacterial growth, and the tissue was analyzed for bacterial invasiveness via Brown-Hopps staining. **(A)** GBS colony counts after 8 hours of infection. Normal bacterial growth, without placental tissue, is shown in purple. Data are presented as box plots (25^th^, 50^th^, and 75^th^ percentiles) with whiskers indicating minimum and maximum values. The ‘+’ symbol within each box denotes the mean. Symbols on the X-axis indicate presence (+) or absence (–) of GBS or LTA. Glucose concentrations are expressed in mM. n=9 independent experiments in triplicate. Kruskal–Wallis followed by Dunn’s multiple comparisons *post hoc* test. Different letters indicate p<0.05. **(B)** Count of syncytial disruption zones by three independent observers. Statistical analysis was performed using ordinary One-way ANOVA followed by Tukey’s *post-hoc*. **(C, E, G)** Histological analysis of GBS-infected villous explants exposed to 5 mM **(C)**, 10 mM **(E)**, or 50 mM **(G)** glucose. Bacteria within the villous trees are visualized in blue. Arrows indicate areas of syncytial degradation. Representative images from three independent experiments. Insets **(C, E, G)** correspond to magnified views shown in **(D, F, H)**, respectively.

After 8 hours of infection, we observed a significant increase in the colony-forming unit (CFU) counts of GBS in the culture media of villous explants pre-exposed to high and severely high glucose concentrations, compared to explants cultured in control medium (**Figure 1A**). Additionally, the bactericidal ability of villous cells was evident when comparing bacterial growth in the absence of explants (1.6 x 10^9^ CFU/mL/g of tissue) to bacterial growth in the presence of normoglycemic explants (1.1 x 10^6^ CFU/mL/g of tissue) (**Figure 1A**).

Next, we examined the morphological changes in villous explants after GBS infection and evaluated whether high glucose conditions affect this response (**Figures 1B–H**). Brown-Hopps staining was used to visualize bacteria, which appeared blue. GBS was primarily contained at the syncytial barrier in all glucose environments. However, higher glucose concentrations significantly disrupted the continuity of the syncytial layer (**Figure 1B**), showing signs of syncytium shedding and destruction of villous integrity in some regions (arrows and magnified insets).

Together, these findings highlight the critical impact of a high glucose environment on both the control of bacterial growth and the integrity of the syncytial barrier, which represents a physical defensive mechanism against GBS in the human placenta.

### 3.2 Hyperglycemia differentially regulated the synthesis of chemokines and cytokines in response to GBS infection

**Figure 2:**
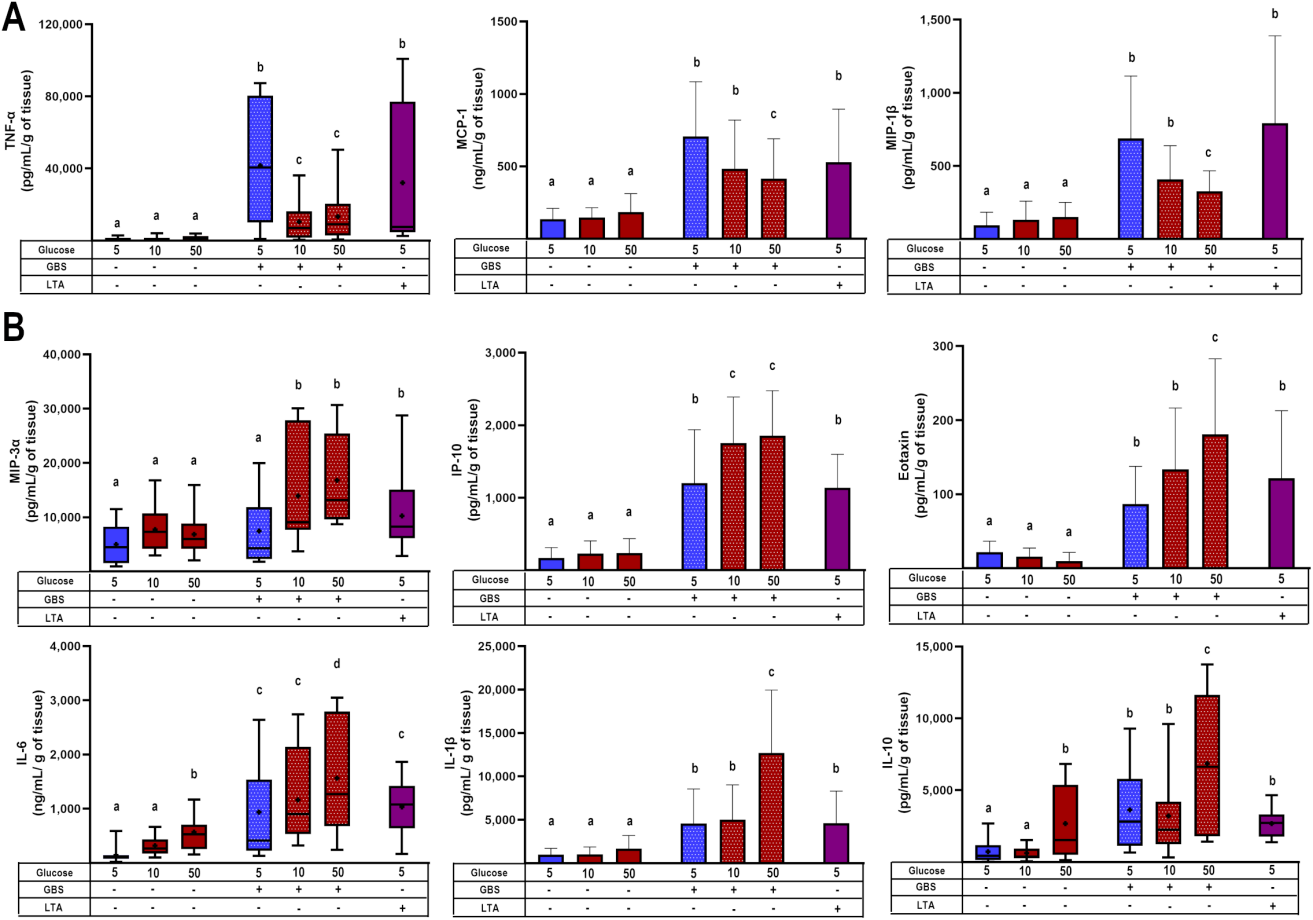
Glucose exposure modulates placental chemotactic and inflammatory responses to GBS infection. **(A)** Pre-exposure to high glucose reduces the GBS-induced secretion of TNF-α, MCP-1, and MIP-1β in villous explants. **(B)** Pre-exposure to high glucose enhances the secretion of MIP-3α, IP-10, eotaxin, IL-6, IL-1β, and IL-10 in GBS-infected villous explants. Data with normal distribution are presented as mean ± SD (column bars), while non-normally distributed data are shown as box plots (25^th^, 50^th^, and 75^th^ percentiles) with whiskers indicating minimum and maximum values. The ‘+’ symbol within each box denotes the mean. Symbols on the X-axis indicate the presence (+) or absence (–) of GBS or LTA. n=5 independent experiments, each performed in triplicate. Statistical analyses were conducted using ordinary One-way ANOVA followed by Tukey’s *post-hoc*, or the Kruskal–Wallis followed by Dunn’s multiple comparisons, as appropriate. Different letters indicate p<0.05. Glucose concentrations are expressed in mM. GBS: Group B Streptococcus (8 h infection at 1x10^5^ CFU/mL). LTA: lipoteichoic acid (5 µg/mL).

The next step was to measure the secretion profile of chemokines and cytokines. TNF-α, MCP-1, and MIP-1β (**Figure 2A**) were primarily induced by GBS infection, but the dual challenge with high glucose significantly reduced their secretion. In contrast, MIP-3α, IP-10, eotaxin, IL-6, IL-1β, and IL-10 (**Figure 2B**) although also induced by GBS infection, were not downregulated by the pre-exposure to high glucose. Instead, the secretion of these cytokines was further increased by the dual inflammatory challenge. This pattern is insightful, as it sheds light on the specific role of each molecule in modulating the immune response. For instance, as MCP-1 and MIP-1β are closely involved in recruiting monocytes and NK cells, our results highlight the repressive effect of hyperglycemia upon the chemotactic activity of these cytokines/chemokines. Another interesting result was IL-10 secretion in the absence of GBS infection. As depicted in **Figure 2B**, IL-10 was the only cytokine significantly upregulated by 50 mM glucose. Considering the anti-inflammatory role of this cytokine, its stimulation by high glucose might explain the absence of an increase in the secretion of inflammatory molecules under this condition as early as 8 h.

We also quantified the secretion of additional chemokines (IL-8, I-TAC, MIG, MIP-1α, TARC) and cytokines (IL-12p70, IL-17a, IL-18, IL-23, IL-33, IFN-α2), which were exclusively upregulated by GBS infection but were not further modulated by high glucose (**Supplementary Figure 3**).

**Figure 3:**
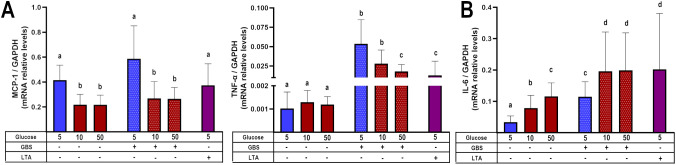
Glucose exposure modulates placental inflammatory gene expression in response to GBS infection in a chemokine- and cytokine-dependent manner. Pre-exposure to high glucose reduces the gene expression of MCP-1 and TNF-α **(A)**, while enhancing IL-6 gene expression **(B)** in GBS-infected villous explants. Data are presented as mean ± SD (column bars). Symbols on the X-axis indicate the presence (+) or absence (–) of GBS or LTA. n=4 independent experiments performed in triplicate. Statistical analyses were conducted using ordinary One-way ANOVA followed by Tukey’s *post-hoc*. Different letters indicate p<0.05. Glucose concentrations are expressed in mM. GBS: Group B Streptococcus (8 h infection at 1x10^5^ CFU/mL). LTA: lipoteichoic acid (5 µg/mL).

To corroborate the previous findings, we evaluated the regulation of some chemokines and cytokines at the transcription level. We observed that the dual stimulus of infection and hyperglycemia downregulated the gene expression of MCP-1 and TNF-α (**Figure 3A**), consistent with the results from the secretion assay. In contrast, IL-6 was upregulated under the dual stimulus compared to explants cultured in control glucose media and infected with GBS (**Figure 3B**). Notably, in the absence of GBS, the gene expression of MCP-1 was significantly downregulated by high glucose, while that of IL-6 was upregulated (**Figures 3A, B**). Finally, GBS infection increased IL-1β and IL-8 mRNA levels, but these were not further modulated by high glucose concentrations (**Supplementary Figure 4**), in a similar pattern as the results from the secretion assay.

These results confirm that the combined stimulus of hyperglycemia and GBS infection led to a chemokine/cytokine-specific pattern in human villi, leading to an inflammatory chemokine imbalance.

### 3.3 Hyperglycemia and GBS infection downregulate chemotaxis of mononuclear intervillous blood cells

After observing that certain cytokines and chemokines were upregulated in the combined challenge of GBS infection and hyperglycemia (**Figures 2B**, **3B**), while TNF-α, MCP-1 and MIP-1β were downregulated under these conditions (**Figures 2A**, **3A**), we investigated whether the chemoattraction of immune cells would be affected.

**Figure 4:**
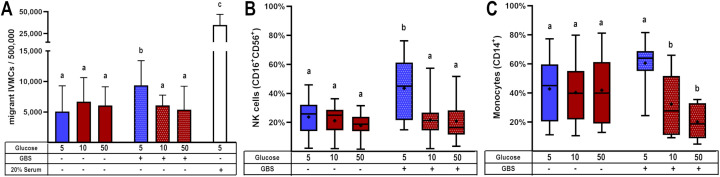
Glucose exposure reduces the chemotactic attraction of mononuclear cells from intervillous blood in response to placental GBS infection. Villous explants were pre-exposed to varying glucose concentrations for 48 h, followed by infection with GBS (1x10^5^ CFU/mL). The resulting culture media were stored at -40°C and later used in migration assays employing the Boyden chamber system. Conditioned media from GBS-infected, glucose-treated villous explants were tested for their ability to induce migration of: **(A)** IVMC, **(B)** NK cells (CD16+CD56+), and **(C)** monocytes (CD14+) as assessed by immunophenotyping via flow cytometry. Data with normal distribution are presented as mean ± SD (column bars), while non-normally distributed data are shown as box plots (25^th^, 50^th^, and 75^th^ percentiles) with whiskers indicating minimum and maximum values. The ‘+’ symbol within each box denotes the mean. Symbols on the X-axis indicate the presence (+) or absence (–) of GBS or LTA. n=4 independent experiments for IVMC migration, and n=8 independent experiments for cell phenotyping, each performed in triplicate. Statistical analyses were conducted using ordinary One-way ANOVA followed by Tukey’s *post-hoc*, or the Kruskal–Wallis followed by Dunn’s multiple comparisons, as appropriate. Different letters indicate p<0.05. Glucose concentrations are expressed in mM. GBS: Group B Streptococcus (8 h infection at 1x10^5^ CFU/mL). 20% of newborn calf serum was used as a positive control for cell migration (4A).

Our results demonstrated that GBS infection enhanced IVMC migration compared to uninfected groups. However, when the villous were cultured under hyperglycemic conditions, IVMC migration decreased significantly (**Figure 4A**). Next, we examined the phenotype of these migrating cells. We found that the cell populations exhibiting reduced migration were monocytes and NK cells (**Figures 4B, C**), which correspondingly are chemoattracted by MCP-1 and MIP-1β.

On the other hand, T lymphocytes and NKT cells did not exhibit migration toward villous media, regardless of high glucose concentrations or GBS infection (**Supplementary Figures 5A, C**). B lymphocytes showed a trend toward decreased migration under the combined stimulus; however, this difference was not statistically significant (**Supplementary Figure 5B**).

### 3.4 Hyperglycemia and GBS infection impair TLR-2 expression

After observing decreased cytokine and chemokine molecules, and migration of total IVMC and specific cells, by both, hyperglycemia and infection, we sought to understand the mechanism underlying this phenomenon. TLR-2 is the main PRR responsible for sensing gram-positive bacteria such as GBS. In addition, TLR-2 signaling triggers NF-κB and regulates the expression of pro-inflammatory cytokines and chemokines (28). Therefore, we decided to study whether its expression at the protein or mRNA level is affected.

**Figure 5:**
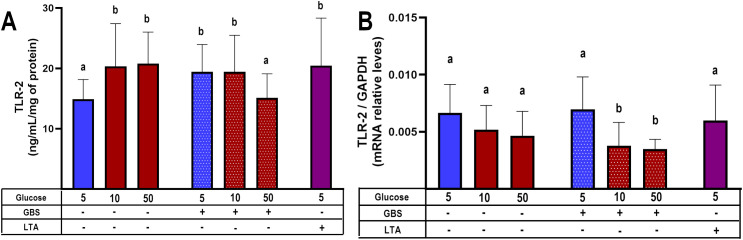
TLR-2 synthesis is down-regulated in GBS-infected villous explants under hyperglycemic conditions. Villous explants were pre-exposed to varying glucose concentrations for 48 h, followed by 8-hour infection with GBS (1x10^5^ CFU/mL). Protein and mRNA were extracted to assess: **(A)** Cellular TLR-2 protein content, and **(B)** Relative TLR-2 mRNA expression levels. Symbols on the X-axis indicate the presence (+) or absence (–) of GBS or LTA. Data are presented as mean ± SD (column bars). n=4 independent experiments performed in triplicate. Statistical analyses were conducted using ordinary One-way ANOVA followed by Tukey’s *post-hoc*. Different letters indicate p<0.05. Glucose concentrations are expressed in mM. GBS: Group B Streptococcus (8 h infection at 1x10^5^ CFU/mL). LTA: lipoteichoic acid (5 µg/mL).

Our results showed that TLR-2 protein levels were significantly induced by high glucose levels and GBS infection. But GBS infection in explants pre-exposed to glucose 50 mM significantly diminished TLR-2 levels (**Figure 5A**). Coincidently, gene expression assay indicated that combination of GBS and high glucose levels diminished TLR-2 mRNA levels (**Figure 5B**).

## 4 Discussion

Pregnancy entails complex immunological adaptations to protect both the fetus and the mother. However, pathological conditions during pregnancy, like GDM, can compromise placental defenses. Clinical and experimental data have linked GDM with increased susceptibility to urinary and cervicovaginal infections (8–10) and altered immune responses, including reduced production of antimicrobial peptides (5, 29–31) and dysregulated chemokine profiles (32).

Building upon our previous findings (5), here we explored how hyperglycemia modulates the placental immune response to GBS. We confirmed that hyperglycemia exacerbates bacterial proliferation and damages the syncytial barrier, likely facilitating vertical transmission (**Figure 1**). Notably, we observed that GBS infection under hyperglycemic conditions led to a selective suppression of key chemokines, including MCP-1, MIP-1β, and TNF-α (**Figures 2A**, **3A**). Also, this chemokine suppression was translated into a reduced chemoattraction of monocytes and NK cells, as shown in our IVMC migration assays (**Figure 4**).

**Figure 6:**
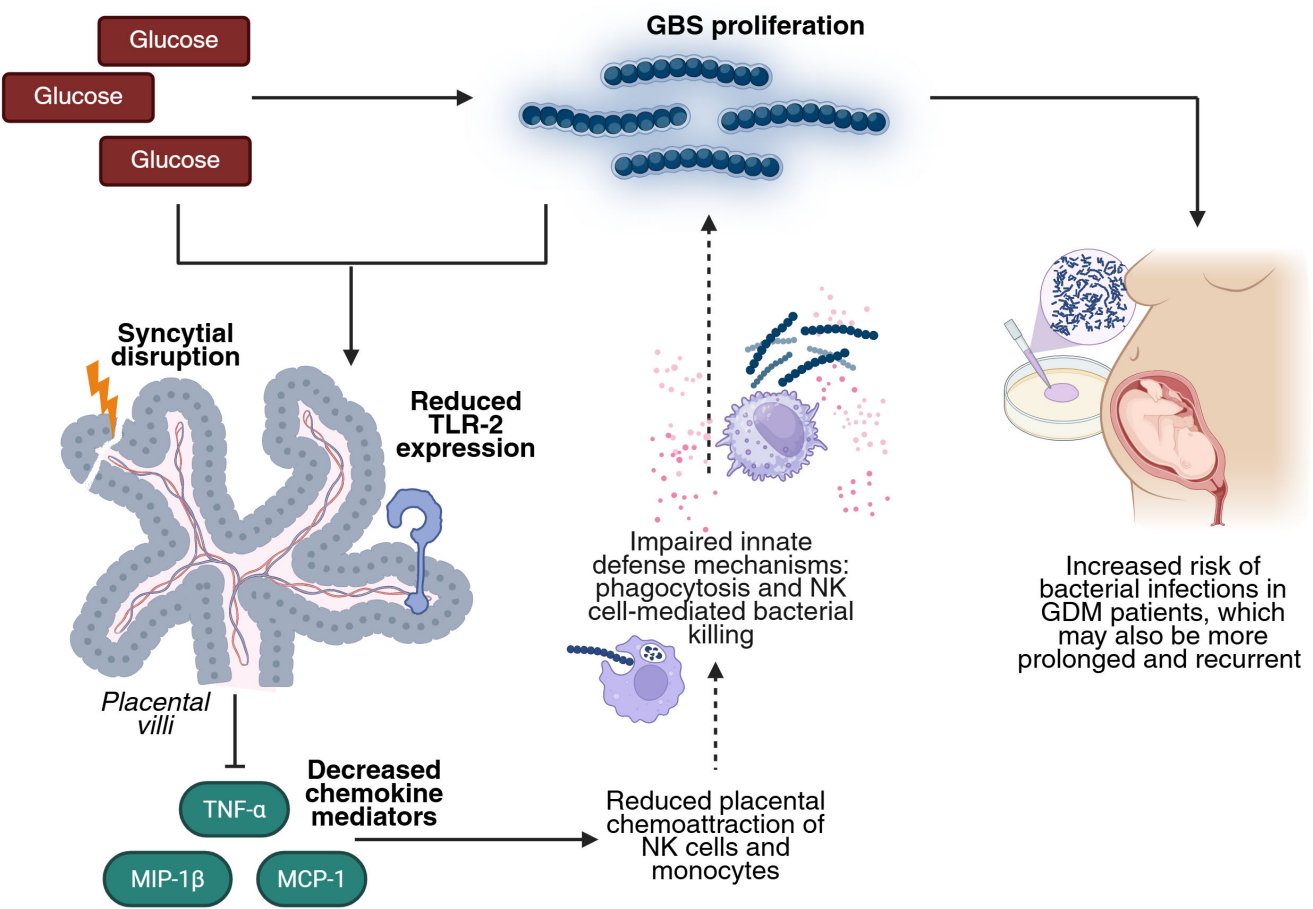
High glucose enhances GBS infection by impairing TLR-2 expression and the chemotactic response in the human placenta. Elevated glucose levels favor GBS proliferation. The combined insult of hyperglycemia and GBS infection reduces placental TLR-2 expression, impairing bacteria detection and downregulating key chemokines (TNF-α, MCP-1, MIP-1β). This leads to diminished recruitment of NK cells and monocytes, which weakens innate defense mechanisms such as phagocytosis and NK cell-mediated killing. This dysregulated environment favors GBS persistence and proliferation, damages the structure of the placental barrier, and increases the risk, duration, and recurrence of bacterial infection in GDM pregnancies. Created in BioRender (https://BioRender.com).

Increased TLR-2 expression has been reported in the placenta of mothers with GDM (33). A systematic review also concluded that diabetes is associated with elevated TLR-2 mRNA and protein levels in peripheral blood, as well as increased receptor activation (34). Conversely, in a murine model of induced diabetes, reduced TLR-2 expression was reported in the brain, kidney, and liver (29). In this study, we found that GBS infection alone upregulated TLR-2 expression in placental explants, but this response was blunted in the presence of high glucose (**Figure 5**). These findings are consistent with those of Panda et al., who reported elevated TLR-2 expression in macrophages from T2DM patients, which notably declined in T2DM patients co-infected with *Mycobacterium tuberculosis* (35). Our results support the hypothesis that hyperglycemia may impair the placenta’s ability to detect GBS via TLR-2, even in the presence of increased bacterial load. This impaired recognition likely contributes to the observed downregulation of key chemotactic mediators — TNF-α, MCP-1, and MIP-1β — observed when both infection and hyperglycemia are present. These chemokines are essential for mounting an effective host response against invading bacteria, as they regulate the trafficking of Th1 cells, basophils, NK cells, monocytes, and macrophages (32). This chemokine imbalance may consequently impair the recruitment of IVMCs, particularly monocytes and NK cells, weakening innate immune responses such as phagocytosis and NK cell-mediated bacterial killing. Then, this immunosuppressive environment may promote bacterial survival, proliferation, and invasiveness, while also compromising local immune control. The increased GBS burden could further damage the syncytial barrier, ultimately undermining the structural integrity of the placenta (**Figure 6**). Notably, damage to the syncytial actin cytoskeleton is a critical mechanism that compromises the placental barrier allowing the pathogen to reach the fetus (36). Bergeron et al., demonstrated in a rat model of GBS-induced chorioamnionitis, that this pathogen induces placental matrix metalloproteinase-10 (MMP-10) release (37), which could be a potential mediator of the syncytial degradation observed in response to infection.

Taken together, these findings indicate that GBS infection causes a distinct reduced inflammatory and chemotactic profile in the context of GDM, compared to normoglycemic conditions. This dysregulated response may help to explain the increased persistence and severity of GBS infection observed in GDM patients (8–11), underscoring their heightened vulnerability to bacterial complications during pregnancy.

An additional mechanism that may contribute to the observed cytokine and chemokine dysregulation is immune tolerization, previously described in macrophages re-exposed to inflammatory stimuli such as LPS or lipoteichoic acid. In this context, re-stimulation leads to suppressed proinflammatory cytokine production, potentially through epigenetic modifications, as a protective strategy to prevent excessive inflammation (38, 39). Similarly, in our model, the dual challenge of hyperglycemia and GBS may trigger a tolerization-like response, selectively downregulating TNF-α, MCP-1, and MIP-1β to avoid fetal harm, yet at the cost of impaired immune defense.

This study has some limitations. While we excluded placentas from women with metabolic disorders, those from overweight women were included due to their high prevalence in the Mexican population (40). Although pregestational overweight has been associated with increased oxidative stress (41), we did not observe enhanced baseline inflammation in our samples. Additionally, while we processed 36 placentas, specific analyses were performed with 4–9 samples, limiting statistical adjustment for potential confounders such as weight gain, placental weight, or infant sex.

We acknowledge that the 50 mM glucose concentration used in some assays exceeds physiological levels. However, our results show that key biological effects — including inflammation, GBS proliferation, syncytial barrier disruption, chemokine dysregulation, impaired leukocyte recruitment, and reduced TLR-2 expression — were already evident at 10 mM.

A key strength of our model is the use of intact placental villous explants, which preserve the three-dimensional architecture and cell-cell interactions of the tissue, providing physiologically relevant insights.

Importantly, both GDM and bacterial infections independently increase the risk of adverse maternal and fetal outcomes both in the short and long term (2–4, 42). When combined — a common clinical scenario — their effects are exacerbated, posing a significantly worse prognosis and heightened vulnerability compared to these conditions in isolation. Despite this, these conditions are often monitored separately, neglecting the complex interactions between glucose metabolism and immune response. Our findings highlight the importance of integrated management strategies in GDM pregnancies complicated by infection, and suggest that hyperglycemia may directly impair placental immune responses, increasing susceptibility to vertical transmission. This work contributes to a better understanding of the metabolic-immune crosstalk at the maternal-fetal interface.

## Data Availability

The datasets generated and analyzed during the current study are publicly available. Data can be accessed at: Jiménez-Escutia, Rodrigo; Olmos-Ortiz, Andrea (2025), “Hyperglycemia Enhances Group B Streptococcus Pathogenicity by Impairing TLR2 Expression and Chemotactic Response in the Human Placenta”, Mendeley Data, V1. https://doi.org/10.17632/mc99w5jhc7.1.
