# Supplementary material for: Hyperglycemia enhances group B Streptococcus pathogenicity by impairing TLR2 expression and chemotactic response in the human placenta
Source: Front Immunol. 2025 Jul 17;16:1610381. doi: 10.3389/fimmu.2025.1610381 (PMC12310592; doi:10.3389/fimmu.2025.1610381)
Supplement: Supplementary file 1 [file DataSheet1.docx]

Supplementary Tables

**Supplementary Table 1. Primers’ sequence and probes for RT-qPCR**

| **Gene ID** | **Accession numbers** | **Upper primer** | **Lower primer** | **Probe number** |
| --- | --- | --- | --- | --- |
| ***GAPDH*** | AF261085.1 | agccacatcgctgagacac | gcccaatacgaccaaatcc | 60 |
| ***TNFA*** | M10988.1 | cagcctcttctccttcctga | gccagagggctgattagaga | 29 |
| ***IL1B*** | NM_000576.2 | tacctgtcctgcgtgttgaa | tctttgggtaatttttgggatct | 78 |
| ***IL6*** | NM 000600.1 | gatgagtacaaaagtcctgatcca | ctgcagccactggttctgt | 40 |
| ***IL8*** | NM_001354840.3 | agacagcagagcacacaagc | atggttccttccggtggt | 72 |
| ***MCP1*** | S71513.1 | tctcgcctccagcatgaaag | cttggggaatgaaggtggct | 40 |
| ***TLR2*** | NM_001318789.2 | cctgtgtgactctccatccc | tgtgacattccgacaccgag | 62 |

Supplementary Figures
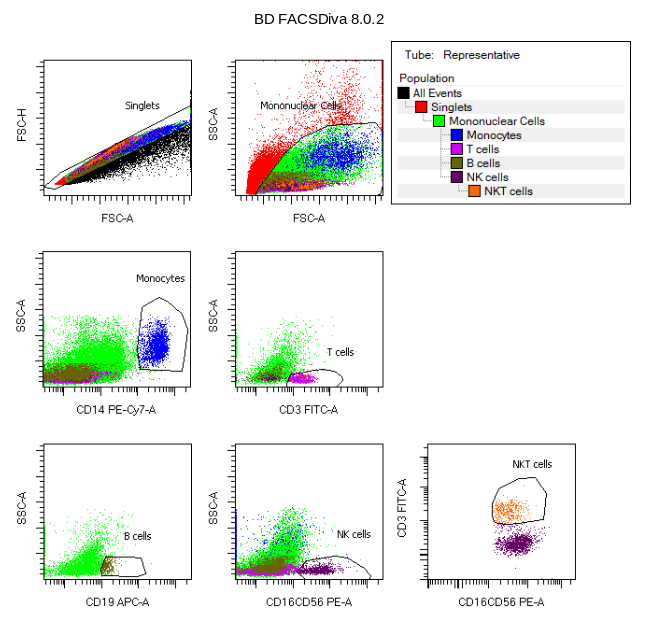


**Supplementary Figure 1. Representative flow cytometry plots illustrating the gating strategy and parameters used for the phenotypic characterization of intervillous blood mononuclear cells (IVMCs).** Initial gating was performed based on forward and side scatter (FSC/SSC) to select mononuclear cells and exclude debris and non-lymphoid events. Subsequent identification of immune cell subsets was carried out according to surface marker expression: CD14+ for monocytes, CD3+ for T cells, CD19+ for B cells, CD16+CD56+ for total NK cells, and CD3+CD16+CD56+ (among total NK cells) for NKT cells.

**
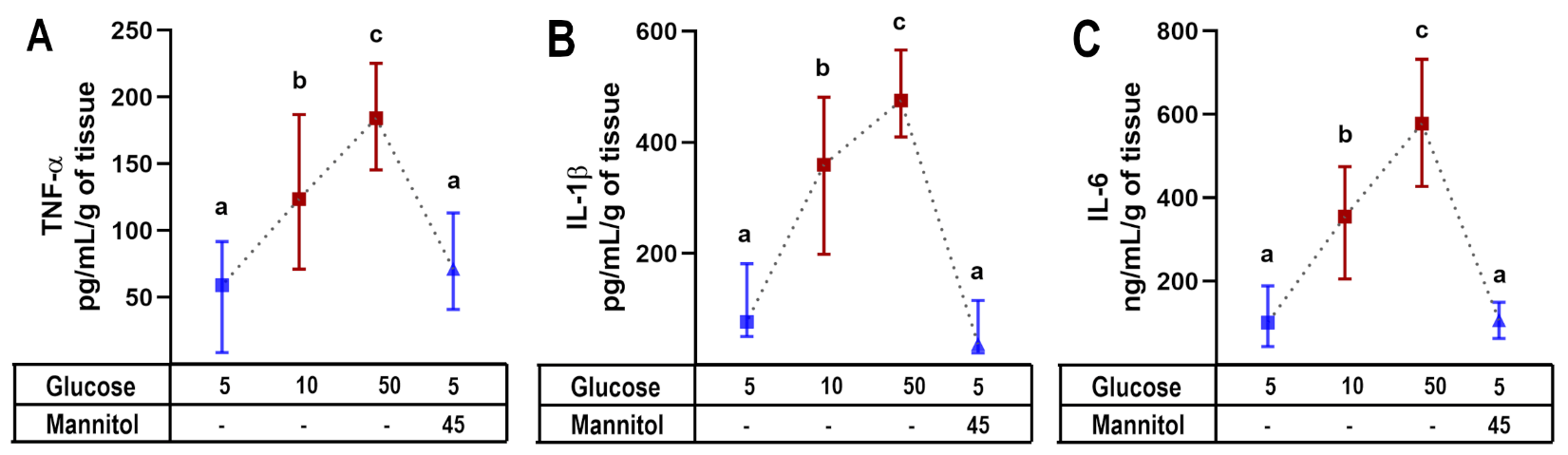
**

**Supplementary Figure 2. Glucose induces a pro-inflammatory response in the human placenta.** Secretion of (A) IL-1β, (B) IL-6, and (C) TNF-α in cultured explants exposed to a glucose curve for 48 h. Data is presented as median and interquartile range. n=6 independent experiments, each performed in triplicate. Statistical analysis was performed using ordinary One-way ANOVA followed by Dunnett’s test for multiple comparisons. Different letters indicate p<0.05. Glucose and mannitol concentration are expressed in mM range.


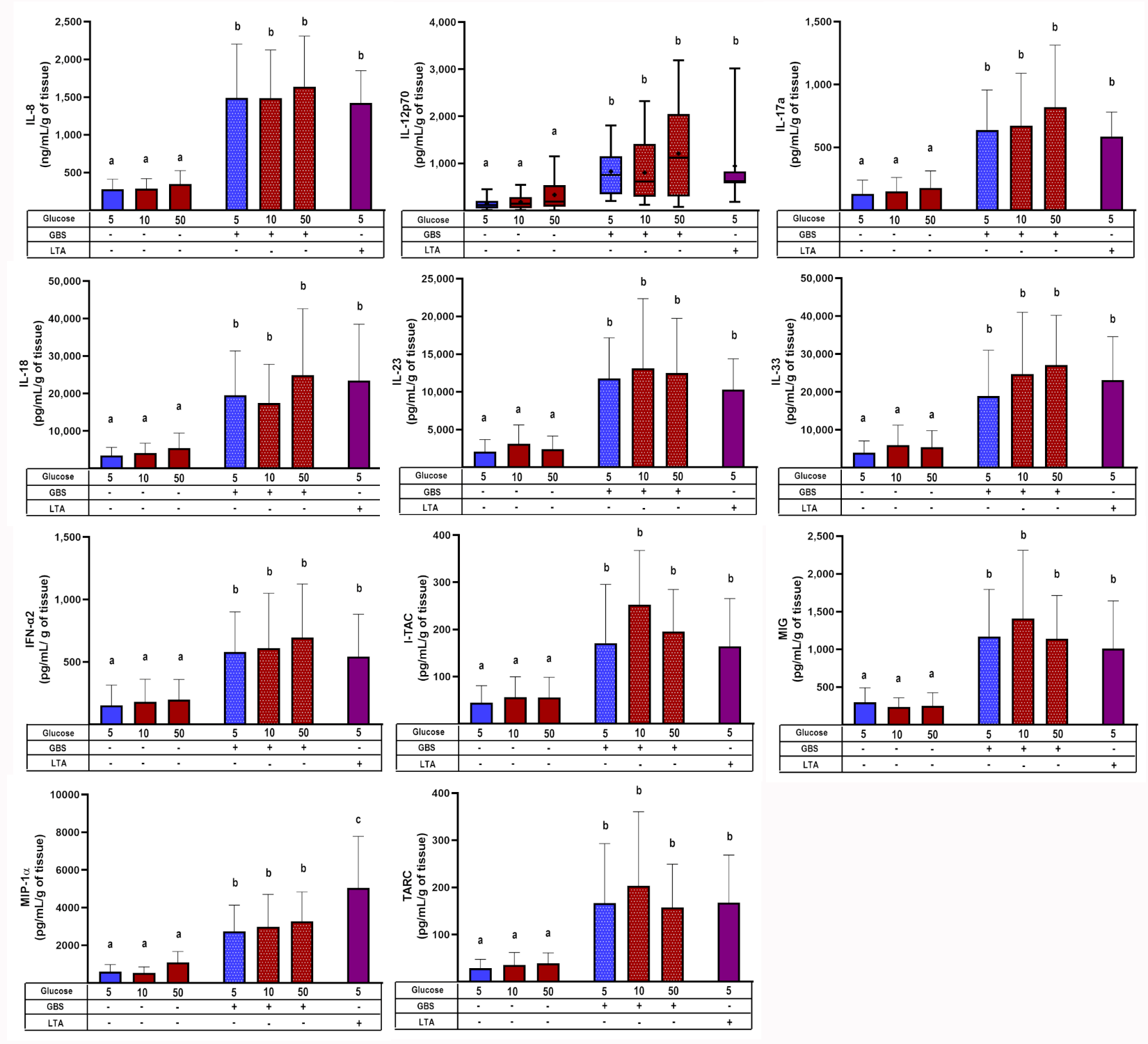


**Supplementary Figure 3.** **GBS infection induces the placental secretion of multiple cytokines and chemokines, but they are not further modulated by pre-exposure to high glucose.** Data with normal distribution are presented as mean ± SD (column bars), while non-normally distributed data are shown as box plots (25^th^, 50^th^, and 75^th^ percentiles) with whiskers indicating minimum and maximum values. The ‘+’ symbol within each box denotes the mean. Symbols on the X-axis indicate the presence (+) or absence (–) of GBS or LTA. n=5 independent experiments, each performed in triplicate. Statistical analyses were conducted using ordinary One-way ANOVA followed by Tukey’s post-hoc, or the Kruskal–Wallis followed by Dunn’s multiple comparisons, as appropriate. Different letters indicate p<0.05. Glucose concentrations are expressed in mM. GBS: Group B Streptococcus (8 h infection at 1x10^5^ CFU/mL). LTA: lipoteichoic acid (5 µg/mL).


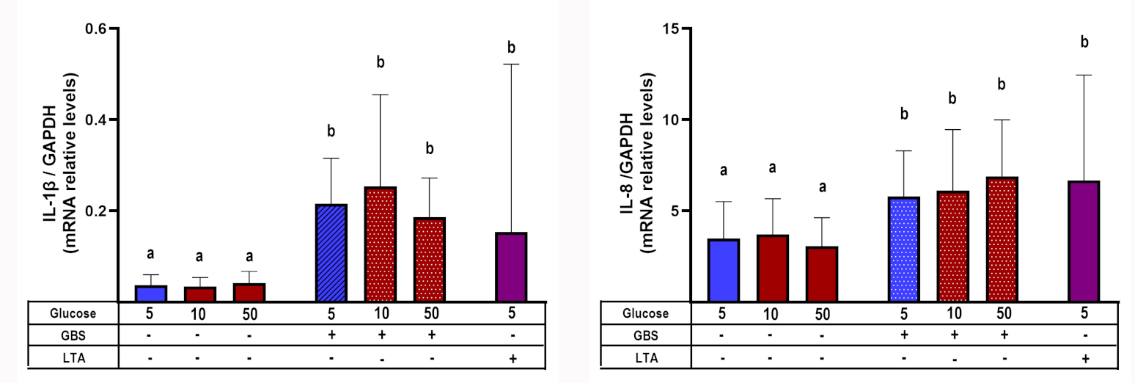


**Supplementary Figure 4.** **GBS infection induces mRNA expression of IL-1β and IL-8, but they are not additionally modulated by pre-exposure to high glucose.** Data are presented as mean ± SD (column bars). Symbols on the X-axis indicate the presence (+) or absence (–) of GBS or LTA. n=4 independent experiments performed in triplicate. Statistical analyses were conducted using ordinary One-way ANOVA followed by Tukey’s post-hoc. Different letters indicate p<0.05. Glucose concentrations are expressed in mM. GBS: Group B Streptococcus (8 h infection at 1x10^5^ CFU/mL). LTA: lipoteichoic acid (5 µg/mL).


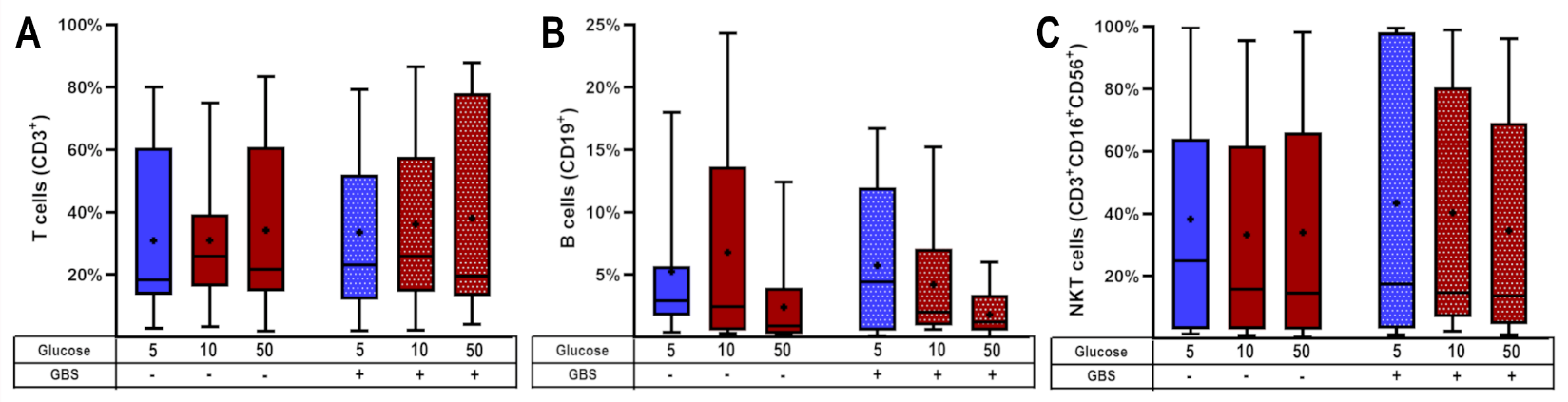


**Supplementary Figure 5.** **Populations of mononuclear cells that** **are not attracted to placental media, either by high glucose concentrations or GBS infection.** Chemotactic capability of placental media for (A) T cells, (B) B cells, and (C) NKT cells. non-normally distributed data are shown as box plots (25^th^, 50^th^, and 75^th^ percentiles) with whiskers indicating minimum and maximum values. The ‘+’ symbol within each box denotes the mean. Symbols on the X-axis indicate the presence (+) or absence (–) of GBS. n=8 independent experiments, each performed in triplicate. Statistical analysis was performed using the Kruskal–Wallis followed by Dunn’s multiple comparisons post hoc test. Glucose concentrations are expressed in mM. GBS: Group B Streptococcus (8 h infection at 1x10^5^ CFU/mL).
